# AID Contributes to Accelerated Disease Progression in the TCL1 Mouse Transplant Model for CLL

**DOI:** 10.3390/cancers13112619

**Published:** 2021-05-26

**Authors:** Maria Schubert, Franz Josef Gassner, Michael Huemer, Jan Philip Höpner, Ekaterina Akimova, Markus Steiner, Alexander Egle, Richard Greil, Nadja Zaborsky, Roland Geisberger

**Affiliations:** 1 Department of Internal Medicine III with Haematology, Medical Oncology, Haemostaseology Infectiology and Rheumatology, Oncologic Center, Salzburg Cancer Research Institute-Laboratory for Immunological and Molecular Cancer Research (SCRI-LIMCR), Paracelsus Medical University, 5020 Salzburg, Austria; ma.schubert@salk.at (M.S.); f.gassner@salk.at (F.J.G.); michael.huemer@posteo.net (M.H.); j.hoepner@salk.at (J.P.H.); e.akimova@salk.at (E.A.); mark.steiner@salk.at (M.S.); a.egle@salk.at (A.E.); r.greil@salk.at (R.G.); n.zaborsky@salk.at (N.Z.); 2 Department of Biosciences, Paris Lodron University of Salzburg, 5020 Salzburg, Austria

**Keywords:** activation induced deaminase (AID), CLL, clonal evolution, deaminase, drug resistance

## Abstract

**Simple Summary:**

Cancers, such as chronic lymphocytic leukemia, frequently acquire consecutive somatic mutations in the genome, which contribute to disease progression and treatment resistance. Activation-induced deaminase is an enzyme responsible for generating the highly diverse B cell repertoire but it can also induce substantial collateral damage within the genome of cells. Hence, it is important to assess whether AID contributes to cancer mutations and to the course of disease. This research shows that AID contributes to the acquisition of somatic cancer-specific mutations in a mouse model for chronic lymphocytic leukemia reflected in prolonged overall survival of leukemic mice lacking AID expression. These data should initiate future studies to assess the effect of AID inhibition on the occurrence of drug resistance.

**Abstract:**

Adaptive somatic mutations conferring treatment resistance and accelerated disease progression is still a major problem in cancer therapy. Additionally in CLL, patients receiving novel, efficient drugs frequently become treatment refractory and eventually relapse. Activation-induced deaminase (AID) is a cytosine deaminase that catalyzes somatic hypermutation of genomic DNA at the immunoglobulin locus in activated B cells. As considerable off-target mutations by AID have been discerned in chronic lymphocytic leukemia, it is essential to investigate to which extent these mutations contribute to disease progression to estimate whether AID inhibition could counteract drug resistance mechanisms. In this study, we examined the TCL1 mouse model for CLL on an AID pro- and deficient background by comparing disease development and mutational landscapes. We provide evidence that AID contributes to the acquisition of somatic cancer-specific mutations also in the TCL1 model and accelerates CLL development particularly in the transplant setting. We conclude that AID is directly determining the fitness of the CLL clone, which prompts further studies to assess the effect of AID inhibition on the occurrence of drug resistance.

## 1. Introduction

Activation-induced deaminase (AID) mediates somatic hypermutation (SHM) and class switch recombination (CSR) of immunoglobulin genes (IGs), which improve affinity and change effector functions of antibodies during humoral immune responses [1,2]. AID achieves these tasks by deaminating cytosines within the genomic Ig locus, thereby generating uracils, which initiate an error-prone repair machinery leading to extensive mutations at the rearranged immunoglobulin variable (IGV) regions (SHM) and DNA breaks at the switch regions, initiating CSR. Although AID activity is tightly regulated, off-target damage outside the Ig-locus occurs and can thus contribute to genome-wide mutations (off-target SHM) and structural variations (off-target CSR), fueling malignant transformation, disease progression and resistance mechanisms [3].

In chronic lymphocytic leukemia (CLL), SHM of the IGV heavy chain (IGHV) of the malignant clone is an important prognostic factor, stratifying patients into CLL with mutated IGHV (CLL-Mut) and with unmutated IGHV (CLL-UM) groups, with CLL-UM having only poor prognosis and shortened overall survival [4]. Strikingly, CLL-UM samples express higher AID levels and AID expression in CLL is an independent unfavorable prognostic factor [5]. Furthermore, mutational signature analysis revealed presence of clustered and genome-wide AID-dependent mutation profiles, which comprise mutations at C within a WRCY/RGYW motif (W=A or T, R=A or G, Y=C or T) throughout the genome, in both CLL-Mut and UM patients [6,7]. From these data, it can be concluded that AID-induced off-target damage significantly contributes to the acquisition of mutations in CLL and hence, the question arises whether inhibition of AID would delay disease onset, decelerate disease progression or synergize with treatment by decreasing mutation rates conferring drug resistance. As no specific clinical AID inhibitors are available so far, we aimed at testing the influence of AID on CLL pathophysiology in TCL1 transgenic mice, a widely used mouse model for CLL-UM [8]. We generated AID deficient TCL1 mice and assessed disease development in primary mice as well as in congenic recipient mice, receiving transplanted leukemic cells from AID proficient or deficient TCL1 animals [9,10]. Furthermore, we applied systematical integrative analyses to decipher mutation spectra, expression profiles and AID-dependent intratumor heterogeneity within IgM switch and VDJ regions in these mouse cohorts. Our results revealed that AID affected disease development in the transplant setting, providing evidence that this mouse model is suitable to determine the impact of AID on drug resistance in future preclinical treatment studies.

## 2. Materials and Methods

### 2.1. Mice

Mouse experiments were performed under the approval of the Austrian animal ethics committee (BMWF 66.012/0009-II/3b/2012, TGV/52/11-2012 and BMBWF-66.012/0002-V/3b/2018). AID knockout TCL1 mice (TCL1-AIDKO) were generated by breeding of TCL1 mice with AID knockout mice [11]. Genotyping was performed on tail tip or ear clip DNA by PCR. The following primers were used for genotyping of TCL1 transgenic mice (309: 5′-AGTGGTAAATATAGGGTTGTCTACACG-3′ and 310: 5′-CCCGTAACTGTAACCTATCCTTTA-3′) and AIDKO mice (for KO 428: 5′-GGCCAGCTCATTCCTCCACT-3′ and 429: 5′-CACTGAGCGCACCTGTAGCC-3′; for wild type 430: 5′-CCTAGTGGCCAAGGTGCAGT-3′ and 431: 5′-TCAGGCTGAGGTTCGGGTTCC-3′).

Adoptive transfer of primary tumors from leukemic TCL1 or TCL1-AIDKO mice was performed by intraperitoneal injection of 10–30 mio splenocytes into C57BL6/J mice and AIDKO^+/−^ C57BL6/J mice. Primary and transplanted (Tx) mice were followed for signs of disease by monthly (primary) and weekly (Tx) tumor load measurements of venous blood samples via flow cytometry (anti-mouse CD19 PE, Clone: 6D5, Cat: 115508; anti-mouse CD4 FITC, Clone: RM4-4, Cat: 116004; anti-mouse CD8a PC7, Clone: 53-6.7, Cat: 552877; anti-mouse CD5 PC5, Clone: 53-7.3, Cat: 100610, BioLegend, San Diego, CA, USA). All mice were sacrificed by CO_2_ suffocation when moribund or presence of >80% CD5/CD19 double-positive CLL cells within the lymphogate in the peripheral blood (representative FACS gating strategy provided in Figure S1). An overview of mice subjected to specific analyses within this study is provided in Table S1, Figure S2 (for TCL1 transgenic mice/tumors) and Figure S3 (for TCL1-AIDKO mice/tumors).

### 2.2. Sorting and DNA and RNA Preparation for Sequencing

Splenocytes of leukemic mice were sorted for CD5/CD19 positive CLL tumor cells using a FACS ARIA III instrument (Becton Dickinson, Franklin Lakes, NJ, USA) as previously described [10]. Germline DNA was extracted from either brain tissue or CD5/CD19 negative splenocytes. DNA was isolated using the DNeasy Blood and Tissue Kit (Qiagen, Hilden, Germany) including RNaseA digestion. RNA was isolated using the High Pure RNA Isolation Kit (Roche, Basel, Switzerland) including DNase digestion.

### 2.3. B Cell Receptor (BCR) Analysis

Libraries for BCR sequencing were prepared as previously described [10]. All libraries were checked for quality and quantity using the Tapestation Bioanalyzer (Agilent, Santa Clara, CA, USA) and the Qubit™ dsDNA HS Assay Kit (Thermo Fisher, Waltham, MA, USA) on a Qubit 2.0 Fluorometer (Invitrogen, Carlsbad, CA, USA). Sequencing was performed on a MiSeq instrument (Illumina, San Diego, CA, USA) using 300 bp paired-end reads. For extraction of the CDR3 amino acid sequences of IGHV, bioinformatics analysis was performed using the MiXCR software (version 3.0.13, https://github.com/milaboratory/mixcr, accessed on 26 May 2021) [12] standard pipeline with export of only productive IGHV clones. For further analysis, a cut-off at 0.01% of total sequencing reads was applied. In addition, whole sequences were extracted into fasta format for analysis of the IGHV mutation status via the IMGT/V-QUEST tool [13]. BCR sequencing data are deposited in the Sequence Read Archive, NCBI, NIH (BioProject: PRJNA475208; SRA accession code SRP150049 and BioProject: PRJNA725403). 

### 2.4. Whole Exome Sequencing (WES)

DNA was subjected to whole-exome library preparation using the SureSelect Mouse All Exon Kit (Agilent, Santa Clara, CA, USA). Libraries were sequenced on a NextSeq instrument (Illumina, San Diego, CA, USA) with 100 bp paired-end reads and a target mean coverage depth of 100×. WES data are accessible in the Sequence Read Archive, NCBI, NIH (BioProject: PRJNA475208; SRA accession code SRP150049 and BioProject: PRJNA725403).

### 2.5. Mutation Analysis

Mutation analysis was performed as previously described with some deviation [10]. In brief, mapping of sequencing reads to mouse reference genome (UCSC mm10) was performed using Burrows–Wheeler aligner with default settings (BWA-MEM v0.7.15, https://github.com/lh3/bwa, accessed on 26 May 2021) [14]. PicardTools (v2.2.2, http://broadinstitute.github.io/picard/ accessed on 20 April, 2016,) with default parameters was used for duplicate removal. Preprocessing of alignments by local realignment around indels and base quality recalibration was performed using Genome Analysis Tool Kit (GATK v3.7) (all with default parameters) [15]. Mpileup file generation was performed using samtools (v1.3.1) (options –B –q 1) [16] followed by somatic variant calling (min-coverage-normal 5, min-coverage-tumor 5, min-var-freq 0.05, somatic-p-value 0.05, strand-filter 1) and filtering of high confidence calls with VarScan2 (v2.4.2) according to Basic Protocol 2 published by Koboldt et al. [17]. Variant annotation was performed using ANNOVAR (version 14 December 2015) [18]. The following annotated mutation classes were considered for further analysis: downstream, exonic, intronic, ncRNA_exonic, splicing, upstream and UTR3/5). Annotated mutations were manually checked for accuracy using Integrative Genomics Viewer (IGV) and the respective germline and tumor line bam files [19,20]. Additionally, all called mutations were manually checked in samples of the same tumor line, compared to other tumor lines, and called if the variant allele frequency was above 5% and the variants were no technical artifacts.

### 2.6. CNV Analysis

Copy number variation (CNV) analysis was performed as previously described with some deviation [10]. In brief, depth of coverage was calculated for each exome target region using Genome Analysis Toolkit (GATK, version 3.8.0) DepthOfCoverage. Coverage data were further analyzed with the R package ExomeCNV (version 1.4, https://cran.r-project.org/src/contrib/Archive/ExomeCNV/, accessed on 26 May 2021) using the Circular Binary Segmentation method (CBS) to combine genomic bins into regions of equal copy number. As germline and primary tumor samples of TCL1 transgenic mice were already sequenced in a previous run [10], a pooled germline reference of samples subjected to the same library preparation (mouse IDs 111_114, 82_114, 91_114, C38) was used for the detection of CNV in samples where no paired germline sample from the same library preparation was available for TCL1 Tx mice. The used references (paired or pooled) are listed in Table S2. For CNV analyses with a pooled reference, segments on the same chromosome were manually combined if the distance between two subsequent segments was less than 0.7 Megabases.

### 2.7. Mutational Signature Analysis

For mutational signature analysis, all annotated and manually checked mutations were transferred into maf file format and analyzed using the “maftools” R package (v2.2.20) [21]. A mutation signature matrix was generated using the function “trinucleotideMatrix” with the “BSgenome.Mmusculus.UCSC.mm10” database as a reference. Mutation signatures were estimated, extracted and compared to the COSMIC database “legacy” (v2 March 2015; https://cancer.sanger.ac.uk/cosmic/signatures_v2.tt, accessed on 20 July, 2020) following the authors’ recommendations to determine cosine similarities against 30 COSMIC signatures for comparison. 

For mutational signature analysis the following mouse samples were assigned to the 5 sample groups: TCL1 group with D22, E31, 347_20, C25, F3, 212 and 221 (WES data already published in Zaborsky; [10]; TCL1 Tx group with CD92, CD95, 702, 703, P42 and P43 (WES data newly generated); TCL1 multiple Tx groups (tumors with >1 rounds of consecutive transplantations) with 642, Q67, R62, Q76 and Q82 (WES data already published in Zaborsky; [10]; TCL1-AIDKO group with 111_114, 82_114 and 91_114 (WES data newly generated); and TCL1-AIDKO Tx group with 72_14, 73_14, 121_14, 122_14 and 125_14 (WES data newly generated). Detailed information on group composition and the corresponding tumor lines are shown in Table S1, mutations included in mutational signature analysis are listed in Table S3.

### 2.8. RNAseq

All RNAs were checked for integrity and quantity using the Tapestation Bioanalyzer (Agilent, Santa Clara, CA, USA) and the Qubit RNA HS Assay Kits (Thermo Fisher, Waltham, MA, USA) on a Qubit 2.0 Fluorometer (Invitrogen, Carlsbad, CA, USA). RNA libraries were generated using the NEBNext Ultra II Directional RNA Library Prep Kit for Illumina with 150 ng input RNA and the NebNext Poly (A) mRNA magnetic isolation module (NEB, Ipswich, UK). Library preparation was performed according to the manufacturer’s instructions (NEBNext^®^ UltraTM II Directional RNA Library Prep Kit for Illumina^®^, Instruction Manual version 1.0, April 2017). Sequencing was performed on an Illumina NovaSeq platform (Illumina, San Diego, CA, USA) with 100 bp paired-end reads.

Integrity and sequencing quality of the output fastq files was evaluated using FastQC (v0.11.5) [22]. Sequencing reads were aligned using the STAR aligner (v2.7.3a) [23] and refGene-annotated mm10 genome. Read summarization to mm10 refGene exons was performed using “featureCounts” (subread-1.6.3 package) with a minimal overlap of 60 bases [24]. Differential RNA expression analysis was calculated using the R package edgeR (v3.26.8) [25]. In brief, genes with no or low read counts were filtered (filterByExpr) and a normalization factor was calculated for each sample (calcNormFactors). A multidimensional scaling (MDS) plot was generated to approximate log2 fold changes between samples on a two-dimensional scale (plotMDS). Gene-wise exact test for differences in the means between two groups was computed. Overrepresentation (OR) analysis of deregulated genes and gene set enrichment analysis (GSEA) of Gene Ontology (GO) and KEGG annotation databases were performed using the “clusterProfiler” R package (v3.12.0, nperm = 10,000 and GSSize 3-800 for GO, nperm = 1000 and minGSSize = 120 for KEGG) [26] and simplified to collapse overlapping gene sets (clusterProfiler: simplify, cutoff = 0.7). Significant results were visualized using “enrichplot” (v1.4.0) [27] or “pheatmap” (v1.0.12, CRAN). “Pathview” (v1.24.0) [28] was used for pathway mapping. Multiple testing correction algorithm, “fdr” and a cutoff of 0.05 was used to determine statistical significance in all analyses. RNAseq data are accessible in the Sequence Read Archive, NCBI, NIH (BioProject: PRJNA725403). RNAseq metrics are provided in Table S4.

### 2.9. Sµ Region Sequencing

40 ng genomic DNA of sorted CLL cells was subjected to PCR amplification of a part of the Sµ region (chr12: 113425468-113426926; mm10). As a positive control, CH12F3 cells (a kind gift from Rushad Pavri, Vienna, Austria) were stimulated for 72 h with anti-CD40 (1 µg/mL), IL4 (5 ng/mL) and TGFß1 (0.5 ng/mL) to induce CSR prior DNA isolation [29]. Tail DNA of a primary TCL1 mouse (314_20) was used as a negative control. The following primers were used for amplification: RG526 5′-agtcagtgacgtgaagggcttctaag-3′ (fwd) and RG527 5′-gctactccagagtatctcatttcagatc-3′ (rev) in a similar manner to that described previously [30,31]. After subsequent agarose gel electrophoresis, the desired DNA fragment (1459 bp) was extracted using NucleoSpin Gel Clean-up Kit (Macherey-Nagel, Düren, Germany) according to manufacturer’s protocol. In order to increase DNA quality, determined by 260/280 and 260/230 ratios measured on NanoDrop, an additional bead purification step was performed using AMPure XP Beads (A63881, Beckman Coulter, Brea, CA, US). Next, the samples were prepared for sequencing with Nextera XT DNA Library Preparation Kit (FC-131-1024, Illumina, San Diego, CA, USA) according to manufacturer’s instructions. In brief, 1 ng of large PCR product (described above) was fragmented applying a transposome based technique, resulting in 300 bp DNA fragments, indexed (Nextera XT Index Kit v2, Set A, FC-131-2001, Illumina, San Diego, CA, USA) and bead purified. Subsequently, all libraries were checked for quality and quantity using the Tapestation Bioanalyzer (Agilent, Santa Clara, CA, USA) and the Qubit™ dsDNA HS Assay Kit (Thermo Fisher, Waltham, MA, USA) on a Qubit 2.0 Fluorometer (Invitrogen, Carlsbad, CA, USA) and the average fragment size was determined. Libraries were sequenced on a MiSeq instrument (Illumina, San Diego, CA, USA) with 300 bp paired-end reads and a target mean coverage depth of 20,000×.

### 2.10. Sµ Region Mutation Analysis

Fastq files were trimmed using trimmomatic (v0.33, ILLUMINACLIP: TruSeq3PE_adapters: 2:30:10, MINLEN: 100) [32] and aligned to a 1859 bp reference sequence of the Smu region using BWA MEM (v0.7.12-r1039) [14]. As a reference, the amplified region of 1459 bp plus 200 bp at the 3′ and 5′ ends was used covering chr12:113425268-113427126 (mm10). Bam files were processed using PicardTools (v2.22.4, http://broadinstitute.github.io/picard/ accessed on 28 April, 2020,) MarkDuplicates with default parameters. Mpileup files were generated using samtools (v1.5, –B –q 1) [16] and variants were called using VarScan2 (v2.4.4) mpileup2snp (–min-var-freq 0.001) [33]. Mutational sequence context was extracted using a custom R script. In brief, reference sequence 2 bases up- and downstream of each variant position was extracted from the 1859 bp reference sequence. The sequence context of each G variant was reverse complemented and WRCY, WRC and CG motifs were counted. Sµ sequencing data are accessible in the Sequence Read Archive, NCBI, NIH (BioProject: PRJNA725403). Sµ sequencing metrics are provided in Table S5.

### 2.11. Statistical Analysis and Visualization

Bioinformatics analysis was performed as described above. Heatmaps of somatic mutations were generated in R using “pheatmap”. All other graphs were created in GraphPad Prism 8 or 9 and Inkscape v0.92.3. If not stated differently, statistical analyses were performed in GraphPad Prism 8 or 9 using the following tests: log-rank (Mantel-Cox) tests for survival data and Mann–Whitney tests for group comparison of somatic and Sµ region mutation data. For clonal switch analysis of BCR data, a change in clonal distribution was considered as a clonal switch if the percentage of the major clone in transplanted mice changed by more than 50% of the original major clone fraction in the primary tumor. Clonal switch events were evaluated using Barnard’s unconditional test for significance for 2 × 2 contingency tables in R [34,35].

## 3. Results

### 3.1. Mutation Analysis in TCL1 Mice

**Figure 1:**
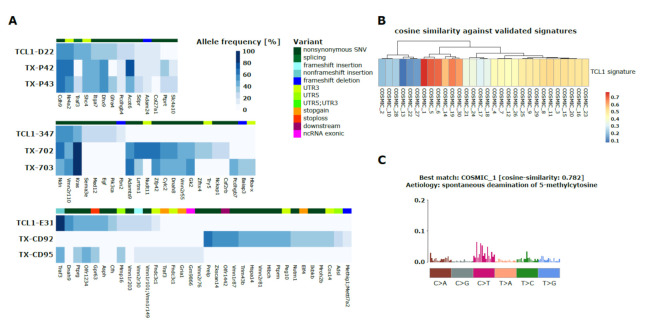
Somatic mutations and mutational signature in TCL1 transgenic mice (TCL1) and congenic recipient mice (TCL1 Tx). (**A**) Heatmaps of somatic mutations found by WES of splenic CLL cells of three TCL1 tumor lines (D22, 347, E31) [10] each transplanted into 2 congenic recipient mice (TCL1 Tx). Color scheme corresponds to allele frequencies of mutations. Different classes of somatic mutations (variant) are depicted as color scheme above the heatmap, gene names are shown below. Synonymous exonic mutations and intronic mutations are not depicted. (**B**) Heatmap showing the cosine similarities of the dominant mutational TCL1 signature found in our cohorts of TCL1 (*n* = 7), TCL1 Tx (*n* = 6) and TCL1 multiple Tx (*n* = 5) samples against 30 validated COSMIC signatures. (**C**) Illustration of COSMIC signature 1, which matches the identified TCL1 signature best (cosine similarity: 0.782) and is dominated by C>T transitions attributed to aging-related deamination of methylated CpGs. The bar plot shows the relative frequencies of the 96 possible substitutions defined by the base substitution (C>A, C>G, C>T, T>A, T>C, T>G) displayed in different colors below the plot and the sequence context of the 3′ and 5′ base next to the base substitution. Each vertical bar represents the percentage of mutations attributed to a specific base substitution within a specific sequence context.

To test the influence of AID on CLL pathophysiology, we first analyzed whether we are able to detect AID-dependent mutation profiles in the TCL1 mouse model. We performed WES of sorted CLL cells from 3 primary TCL1 mice at humane endpoints. In addition, we transplanted these CLLs into 6 congenic wild type (wt) recipient mice (TCL1 Tx), which are fully immune-competent and performed WES upon their outgrowth in the recipients. In line with our previous report, we found a set of non-synonymous mutations for each mouse and we observed that mutation loads increased upon transplantation (Figure 1A and Table S3) [10]. From these WES data (and from our previously published TCL1 WES data comprising further CLL samples obtained after multiple transplantation rounds (TCL1 multiple Tx) from repositories; Sequence Read Archive, NCBI, NIH, BioProject PRJNA475208; SRA accession code SRP150049) we performed mutation signature analysis, a bioinformatics algorithm revealing which mutational processes and aetiologies were operative during the course of cancer development [36]. Using this analysis, we could extract a dominant mutational signature from our cohorts of TCL1, TCL1 Tx and TCL1 multiple Tx samples. This signature was dominated by C>T transitions, particularly defined by C>T mutations at NpCpG trinucleotides. Next, we attributed this signature (TCL1 signature) using cosine similarity to any of the 30 previously described COSMIC signatures, defined by Alexandrov and coworkers [36]. Thereby, we found that the prevailing TCL1 signature had highest similarity with COSMIC signature 1 (cosine-similarity: 0.782), which is attributed to aging-related deamination of methylated CpGs (Figure 1B,C). As COSMIC signature 1 is also the dominant signature in human CLL irrespective of the BCR mutation status [36], our analysis provides the first evidence that a similar main mutational process is operative in the TCL1 mouse model for this disease. However, although mutations at the preferred AID target motif WRCY/RGYW were of rather low relative frequency (Figure S4A,B), we speculated that a portion of the observed mutations at NpCpG could be attributed to AID mediated C>T deaminations, particularly as almost all mutations at CpG sites were C>T transitions (Figure S4C,D). This assumption was strengthened by the observation that AID-dependent mutations were discerned at methylated CpGs in many cancers [37].

### 3.2. CLL Development in AID Deficient TCL1 Mice

**Figure 2:**
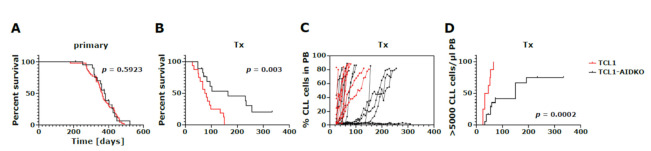
Survival and disease progression of primary and transplanted congenic recipient mice. Overall survival of (**A**) primary tumors with median survival of 363 days in TCL1 mice (*n* = 44) and 375 days in TCL1-AIDKO (*n* = 25) (log-rank (Mantel–Cox) test: *p* = 0.5923) and (**B**) transplanted recipient mice with median survival of 78 days in TCL1 Tx (*n* = 16) and 164 days in TCL1-AIDKO Tx (*n* = 18) (log-rank (Mantel–Cox) test: *p* = 0.0030). (**C**) CLL disease progression of transplanted mice with TCL1 Tx (*n* = 10 of 16 mice shown in B, from which we had FACS data from consecutive blood draws) and TCL1-AIDKO Tx (*n* = 16 of 18 mice shown in B, as 2 mice with T cell leukemias were excluded) is depicted as percent CLL cells (defined as CD5^+^/CD19^+^ cells from all lymphocytes as shown in Figure S1) in the peripheral blood (PB). (**D**) Kaplan–Meier analysis of percentage of TCL1 Tx (*n* = 8 of 10 mice shown in C, from which we could determine absolute cell numbers per µl blood) and TCL1-AIDKO Tx (*n* = 18) transplanted mice entering the leukemic phase (defined as mice having > 5000 CD5^+^/CD19^+^ cells/µl peripheral blood) (log-rank (Mantel-Cox) test: *p* = 0.0002).

To test whether AID contributes to the acquisition of somatic CLL-specific mutations, we generated AID knockout TCL1 mice (TCL1-AIDKO) by breeding TCL1 mice with AID knockout mice. We found that CLL development was similar in TCL1 and TCL1-AIDKO mice, with CD5/CD19 double-positive CLL cells appearing in peripheral blood of both cohorts, leading to a median overall survival of approximately one year (Figure 2A). This suggests that likely tumor driving mutations occurred mainly AID independent by age-related spontaneous deamination of CpGs during the long preleukemic latency phase of TCL1 mice. We next tested whether transplantation of TCL1-AIDKO tumors would lead to increased CLL development in congenic recipients, similar to what we previously observed for TCL1 tumors [10]. Surprisingly, transplantation of TCL1 AIDKO tumors revealed markedly prolonged overall survival of recipient mice compared to recipients receiving AID proficient TCL1 tumors (Figure 2B). In total, 5 out of 18 mice receiving TCL1 AIDKO CLL cells did not develop significant CLL loads in peripheral blood, 2 mice developed T cell lymphoma (characterized by extensive T cell expansion in peripheral blood and spleen) and 4 mice showed significantly delayed CLL development, with latency phases longer than 150 days, albeit tumor expansion in peripheral blood occurred at similar kinetics upon disease onset (Figure 2C,D, Figure S3 and Table S1). The remaining 7 TCL1 AIDKO Tx recipient mice showed a CLL development similar to mice receiving TCL1 tumors, with overall survival of around 60 days (Figure 2C,D and Figure S2). From these data, we inferred that AID-dependent mutational processes might occur in TCL1 tumors in the transplant setting, causing accelerated tumor growth.

**Figure 3:**
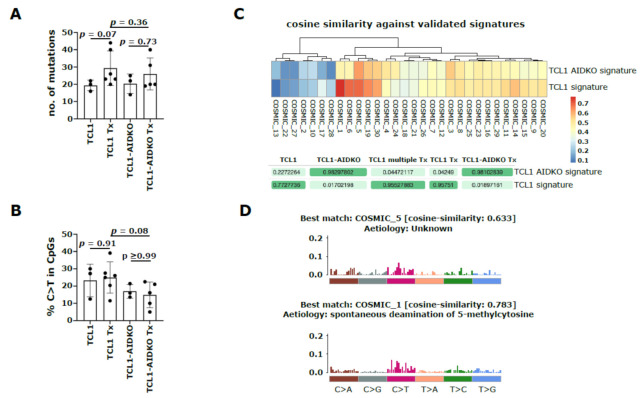
Molecular characteristics and mutational signature of primary (TCL1 and TCL1-AIDKO) and transplanted congenic recipient (TCL1 Tx and TCL1-AIDKO Tx) mice. (**A**) Total number of mutations and (**B**) percentage of C>T transition mutations in CpGs are shown; both two-tailed Mann–Whitney test. (**C**) Heatmap showing the cosine similarities of 2 dominant mutational signatures (TCL1 AIDKO signature and TCL1 signature) found in our cohorts of TCL1 (*n* = 7), TCL1-AIDKO (*n* = 3), TCL1 Tx (*n* = 6), TCL1-AIDKO Tx (*n* = 5) and TCL1 multiple Tx (*n* = 5) samples against 30 validated COSMIC signatures (top). The contribution of each mouse cohort to TCL1 AIDKO signature and TCL1 signature is depicted in percent (bottom). (**D**) Illustration of COSMIC signature 5, which matches the identified TCL1 AIDKO signature best (cosine similarity: 0.633) and has unknown etiology (top). Illustration of COSMIC signature 1, which matches the identified TCL1 signature best (cosine similarity: 0.783) and is dominated by C>T transitions attributed to aging-related deamination of methylated CpGs (bottom). The barplots show the relative frequencies of the 96 possible substitutions defined by the base substitution (C>A, C>G, C>T, T>A, T>C, T>G) displayed in different colors below the plot and the sequence context of the 3′ and 5′ base next to the base substitution. Each vertical bar represents the percentage of mutations attributed to a specific base substitution within a specific sequence context.

To further test this assumption, we analyzed mutations in primary TCL1 AIDKO tumors and TCL1 AIDKO tumors transplanted into congenic recipients by WES (Figure S5 and Table S3). As expected, we observed a trend towards higher mutation loads upon transplantation within the AID proficient group (*p* = 0.07, Figure 3A), whereas the AID deficient tumors had no dramatic increase in mutation load upon transplantation (*p* = 0.73, Figure 3A). Corroboratively, we found fewer transitions at CpGs in TCL1 AIDKO Tx compared to TCL1 Tx samples (*p* = 0.08, Figure 3B and Figure S5). In addition, we found the presence of a distinct mutational signature (TCL1 AIDKO signature) in AID deficient CLL (Figure 3C). This dominant “TCL1 AIDKO signature” had highest similarity to COSMIC signature 5, which is characterized by C>T and T>C mutations, has unknown etiology and displays a transcriptional strand bias for T>C substitutions at ApTpN context (Figure 3D). In line with an altered mutational pattern, AID proficient tumors also showed distinct chromosomal aberrations, particularly a deletion on chr12 close to the IgH region, which could therefore possibly derive from aberrant CSR (Figure S6 and Table S2). In line with these observations, we noted only low AID expression in primary TCL1 tumors by RNAseq analysis, whereas AID mRNA levels were more diverse in the transplanted TCL1 Tx samples with two out of five showing high AID expression (mouse IDs: 113_14 and 120_14), while three samples still had low expression (mouse IDs: 70_14, 71_14 and 703_13 (Figure S7). Strikingly, AID-low samples had shorter survival than the AID-high samples (67, 67 and 81 days vs. 150 and 134 days, Figure S2).

Unbiased analysis of gene expression profiles in the different mouse cohorts revealed 602 deregulated genes (*p* < 0.05) and 8 genes with FDR < 0.05 in the primary TCL1 compared to TCL1 AIDKO tumors (Table S6), whereas between TCL1 and TCL1 AIDKO Tx tumors 1054 deregulated genes (*p* < 0.05) and 17 genes with FDR < 0.05 were found (Table S7). In addition, comparing 3 early progressing to 2 TCL1 AIDKO Tx tumors with delayed disease progression we found 925 deregulated genes (*p* < 0.05) and 19 genes with FDR < 0.05 (Table S8). Performance of overrepresentation (OR) analysis of deregulated genes and gene set enrichment analysis (GSEA) of gene ontology (GO) and Kyoto encyclopedia of genes and genomes (KEGG) annotation databases revealed the GO terms “protein folding chaperone” and “misfolded protein binding” and the KEGG term “spliceosome” to be altered in the TCL1 versus TCL1-AIDKO cohort. Pathway analysis between the TCL1 Tx and TCL1 AIDKO Tx samples could not identify significantly deregulated pathways. Interestingly, pathway analysis between early and late disease onset of TCL1 AIDKO Tx tumors revealed altered “blood vessel development”, “vasculature development”, “cardiovascular system development” and “skeletal muscle cell differentiation” (GO terms) and deregulated “pathways in cancer” (GSEA KEGG terms) (Tables S9 and S10 for OR, Table S11 for GSEA analysis).

**Figure 4:**
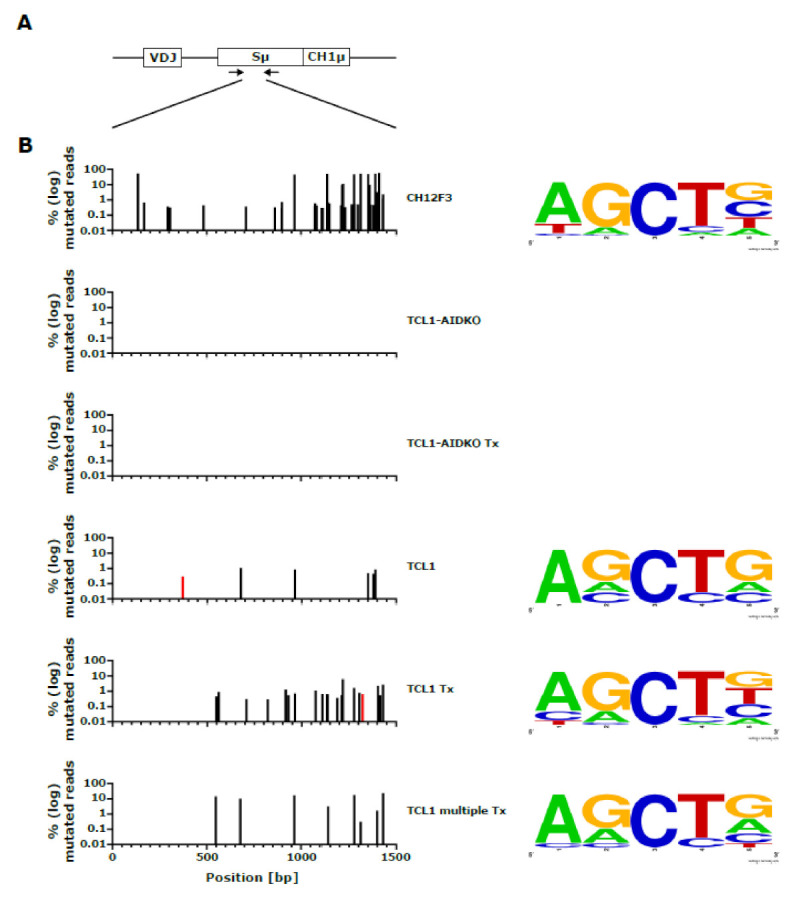

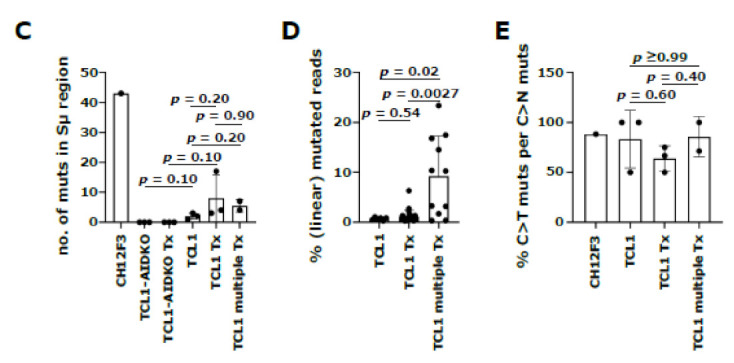
Mutation analysis of Sµ region sequencing of primary (TCL1 and TCL1-AIDKO) and transplanted congenic recipient (TCL1 Tx, TCL1 multiple Tx and TCL1-AIDKO Tx) mice compared to stimulated CH12F3 cells. (**A**) A schematic representation of the IgH locus is indicated with the VDJ gene, the Sμ region, and the first constant exon of the IgM heavy chain (CH1μ). DNA was subjected to PCR to amplify the Sμ region. Primer-binding sites are indicated as arrows. (**B**) The graphs show mutations with a frequency above 0.1% at position 0 to 1500 bp of the amplified Sμ sequence appearing within the sample groups of CH12F3 (*n* = 1), TCL1-AIDKO (*n* = 3), TCL1-AIDKO Tx (*n* = 3), TCL1 (*n* = 3), TCL1 Tx (*n* = 3) and TCL1 multiple Tx (*n* = 2) from top to bottom. The bars indicate percentages of mutated reads per position on a log scale; black bars indicate mutations at C/G, red bars indicate mutations at A/T. On the right side, consensus sequences 2 bases up and downstream of mutated C/Gs are shown per sample group if mutations were present. Consensus sequences were visualized with WebLogo [38] showing the AID target motif WRCY/RGYW as the dominant consensus. (**C**) Total number of mutations in the Sµ region are shown in a two-tailed Mann–Whitney test. (**D**) Percentages of mutated reads over the entire length of the amplifies region are shown on a linear scale; two-tailed Mann–Whitney test. (**E**) Percentage of C>T mutations per number of C>N mutations; two-tailed Mann–Whitney test.

To further validate AID activity in TCL1 tumors, we performed mutation analysis of PCR-amplified IgM switch regions (Sµ region) by NGS-based sequence analysis of sorted CLL cells from primary TCL1, TCL1 Tx, primary TCL1-AIDKO, TCL1-AIDKO Tx (*n* = 3 each) and TCL1 multiple Tx (*n* = 2) samples (Figure 4A). In addition, CH12F3 cells stimulated to induce CSR were analyzed as a positive control [29]. As expected, we found increased mutation rates in this region within the TCL1 cohort, but no mutations in the TCL1-AIDKO cohort (Figure 4B,C). These mutations were characterized by increased frequency upon transplantation (Figure 4D), a dominance of C>T transitions (Figure 4E) and preferentially located within the typical AID WRCY/RGYW motif (Figure 4B). This analysis provides further evidence for AID-dependent mutational activity in the TCL1 cohort compared to the TCL1-AIDKO cohort.

Finally, IGHV sequencing revealed usage of typical CLL-related IGHV genes in the TCL1 and TCL1-AIDKO samples, irrespective of presence of AID, with a dominant major IGHV clone and several minor clones (Figure S8A–C and Tables S12 and S13) [10,39]. However, regarding IGHV mutation status, we found all IGHV clones being unmutated (V region sequence identity > 98%) irrespective of AID expression (Tables S12 and S13). In addition, analysis of transplanted samples revealed that absence of AID resulted in a trend towards higher clonal stability, meaning that dominant IGHV clones in primary samples reappeared in the transplanted tumors, whereas presence of AID yielded expansion of previously minor clones in 3 out 12 transplants (Figure S8D and Table S14).

## 4. Discussion

Here, we provide in-depth analysis of the contribution of AID on disease development in the TCL1 mouse model for CLL. While the acquisition of AID-dependent mutations in human CLL was extracted from previous DNA sequencing approaches [6], the direct impact of AID in the TCL1 mouse model and on the course of disease remained elusive. We found that while absence of AID had no effect in primary TCL1 mice, it resulted in significantly prolonged survival in the transplant setting, pointing to AID mediated off-target mutations in leukemic cells upon expansion in transplanted hosts. This likely indicates extensive tumor-immune crosstalk in healthy mice challenged with malignant cells. This inflammatory setting of immune pressure exerted on CLL cells may evoke transient AID expression [40] contributing to mutagenesis and thereby facilitating outgrowth of a specific subclone. In line with this assumption, we also noticed increased AID expression in some transplanted leukemic cells and a higher clonal plasticity, which means the expansion of a previously minor BCR-specific subclone upon transplantation. Strikingly, AID-low TCL1 Tx samples had shorter survival than the AID-high TCL1 Tx samples, which may indicate that a longer latency could induce AID expression or select for AID-expressing clones, which can thus more easily clonally evolve and expand to overt disease. Consequently, absence of AID could thus explain the occasionally prolonged latency phase and the inability to develop overt leukemia in a subset of knockout tumors. In line with this, we observed high AID-mediated passenger mutations at IgM switch regions exclusively in AID-proficient samples and we noticed selective expansion of clones having such mutations in transplanted tumors. Surprisingly, we did not observe increased SHM at IGHV regions in AID proficient tumors upon transplantation, which could indicate that in contrast to the switch regions, recruitment of AID to IGHV-target sites could be dysregulated.

Strikingly, we observed a mutational signature assigned to aging-dependent deamination of CpG exclusively in AID proficient tumors, corroborating previous reports that AID may contribute to mutations at methylated CpG sites [37]. As AID-mediated deamination of methylated CpGs could lead to their demethylation and thus, to altered gene expression [41,42], we also performed transcriptome analysis from AID pro- and deficient tumors. Indeed, we noticed some gene expression differences between the two cohorts, which could also contribute to the observed delayed disease onset in AID deficient tumors upon transplantation. However, gene pathway analysis did not reveal any significantly deregulated pathways depending on AID expression.

## 5. Conclusions

Summarizing, our study reveals that AID accelerates CLL progression in the TCL1 mouse model in the transplant setting, likely by contributing to off-target mutations. Our results are in line with a recent report showing that constitutive AID overexpression in the TCL1 mouse model shortens overall survival and worsens disease outcome [43]. However, although our data point to increased AID induced mutations in the transplant setting of TCL1 mice, additional impact on non-coding genes (miRNA, lncRNA) or AID dependent epigenetic effects cannot be excluded and should be addressed in future studies [44,45,46]. As the transplant setting induces significant clonal evolution, mimicking selective pressure during therapy, our findings provide a rationale for the development and use of clinical AID inhibitors to prolong treatment effects and minimize relapse rates by preventing AID-induced adaptive mutations. Testing CLL drugs back-to-back in TCL1 and TCL1 AIDKO transplant models will be a valuable tool to identify possible AID sensitive treatment regimens in future studies.

## Data Availability

The data presented in this study are openly available in the Sequence Read Archive, NCBI, NIH (BioProject: PRJNA475208; SRA accession code SRP150049 & BioProject: PRJNA725403).

## Supplementary Materials

The following are available online at https://www.mdpi.com/article/10.3390/cancers13112619/s1, Figure S1: Representative FACS plots, Figure S2: Overview of the transplantation procedure and the TCL1 tumors analysed in this study, Figure S3: Overview of the transplantation procedure and the TCL1-AIDKO tumors analysed in this study, Figure S4: Detailed mutation analysis of TCL1 and TCL1 Tx mice, Figure S5. Somatic mutations in AID knockout TCL1 mice (TCL1-AIDKO) and congenic recipient mice (TCL1-AIDKO Tx), Figure S6: Chromosomal deletion on chr12 in TCL1 tumors, Figure S7: AID expression of sorted tumors from 3 TCL1 and 5 TCL1 Tx mice depicted as counts per million (CPM), Figure S8: IGHV gene segment usage, tumor BCR clonality and clonal switch analysis; Table S1: Mouse characteristics, Table S2: Copy number variations (CNVs), Table S3: Somatic mutation data, Table S4: RNAseq metrics, Table S5: Sµ sequencing metrics, Table S6: Gene expression profiling of primary mice, Table S7: Gene expression profiling of transplanted tumors, Table S8: Gene expression profiling of early vs late TCL1 AIDKO Tx samples, Table S9: Overrepresentation (OR) pathway analysis using gene sets of GO database, Table S10: OR pathway analysis using gene sets of KEGG database, Table S11: Gene set enrichment analysis (GSEA) using KEGG data base, Table S12: BCR analysis of primary mice, Table S13: BCR analysis of transplanted tumors, Table S14: Data input for BCR clonal switch analysis with clonal fractions of the major BCR clone in primary tumors and the same BCR clone in the transplants as well as the percent change upon transplantation and categorization of switch events.
