# Supplementary material for: AID Contributes to Accelerated Disease Progression in the TCL1 Mouse Transplant Model for CLL
Source: Cancers (Basel). 2021 May 26;13(11):2619. doi: 10.3390/cancers13112619 (PMC8198502; doi:10.3390/cancers13112619)
Supplement: Supplementary file 1 [file cancers-13-02619-s001.zip › Appendix supporting figures_new 26 04 2021.pdf]

Appendix: Supplementary figures

Supplementary figure 1

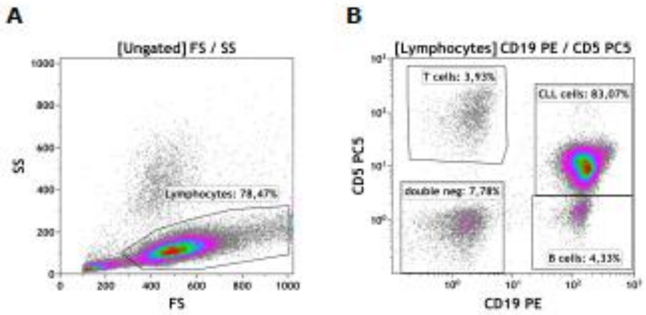

Figure S1: Representative FACS plots. The gating strategy for tumor load measurements of venous blood samples via flow cytometry is shown for lymphocytes (A) and CLL cells, B cells, T cells and CD5/CD19 double negative cells (B).

Supplementary figure 2

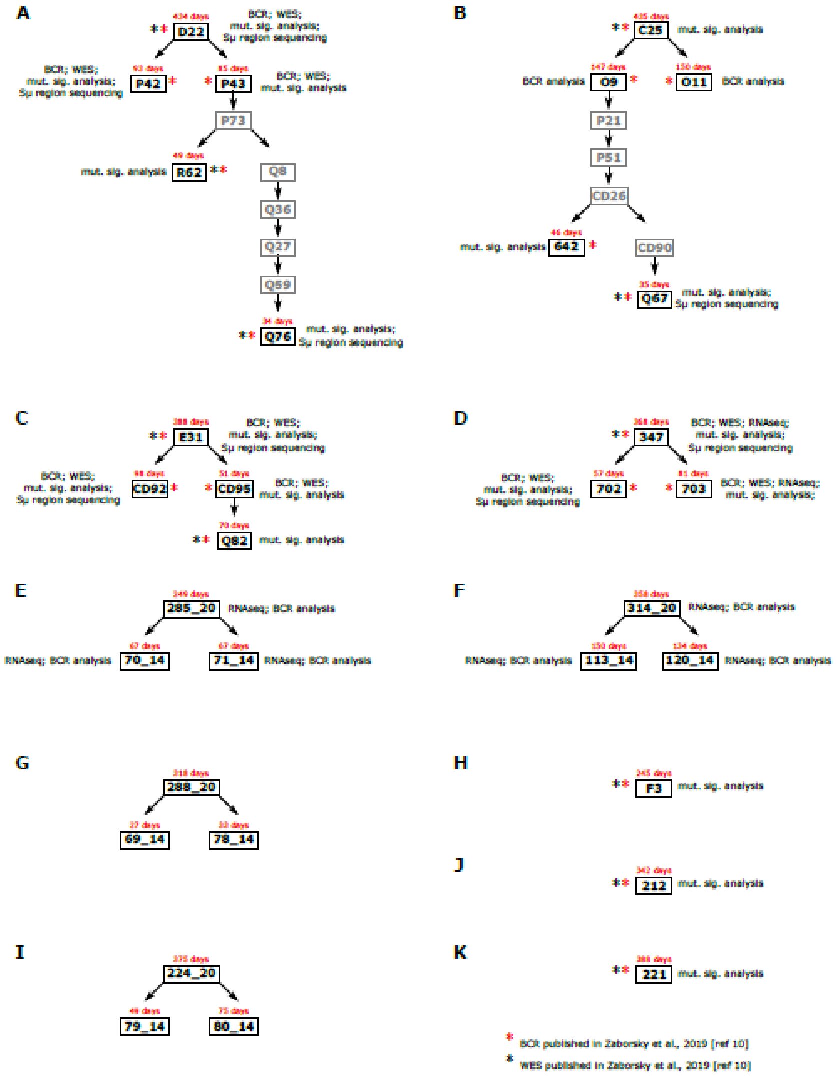

Figure S2: Overview of the transplantation procedure and the TCL1 tumors analysed in this study. TCL1 tumor lines D22 (A), C25 (B), E31 (C), 347 (D), 285_20 (E), 314_20 (F), 288_20 (G), F3 (H), 224_20 (I), 212 (J) and 221 (K) are depicted with mouse IDs in boxes. Transplantation into the recipients is indicated by arrows. Black boxes indicate mice analyzed in this study, grey boxes indicate intermediate recipients not analyzed in this study. Survival times in days are shown above each mouse ID. NGS analyses performed for the present manuscript are shown beside each mouse ID. NGS data already published in Zaborsky et al., 2019 [ref 10] for the respective mouse are displayed by black asterisk for WES data and red asterisk for BCR data.

Supplementary figure 3

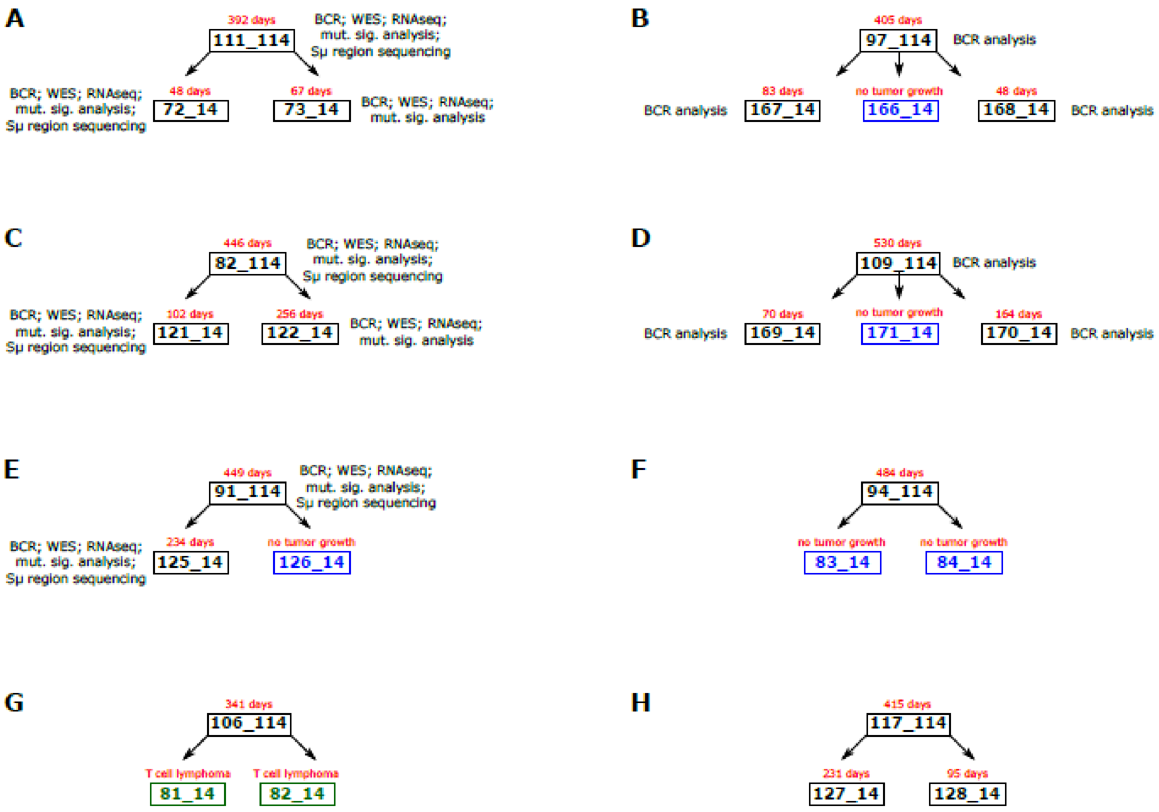

Figure S3: Overview of the transplantation procedure and the TCL1-AIDKO tumors analysed in this study. TCL1-AIDKO tumor lines 111_114 (A), 97_114 (B), 82_114 (C), 109_114 (D), 91_114 (E), 94_114 (F), 106_114 (G) and 117_114 (H) are depicted with mouse IDs in boxes. Transplantation into the recipients is indicated by arrows. Black boxes indicate mice analyzed in this study, blue boxes indicate mice with no tumor development, green boxes indicate mice developing T cell lymphoma. Survival times in days are shown above each mouse ID. NGS analyses performed for the present manuscript are shown beside each mouse ID.

Supplementary figure 4

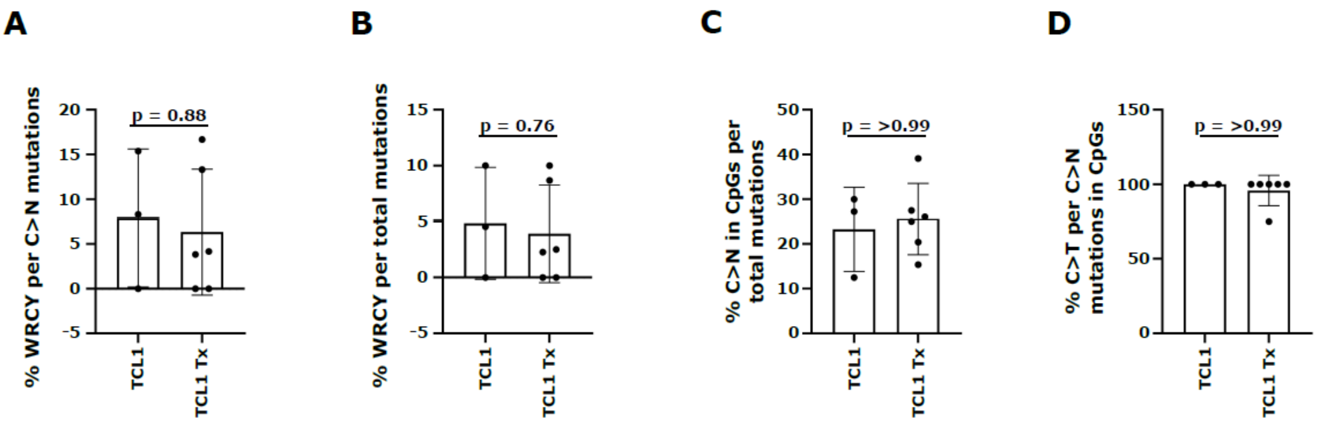

Figure S4: Detailed mutation analysis of TCL1 and TCL1 Tx mice. (A) Percentage of mutations in WRCY motifs per number of C>N mutations. (B) Percentage of mutations in WRCY motifs per total number of mutations. (C) Percentage of C>N mutations in CpGs per total number of mutations. (D) Percentage of C>T mutations per number of C>N mutations in CpGs; two-tailed Mann-Whitney test.

Supplementary figure 5

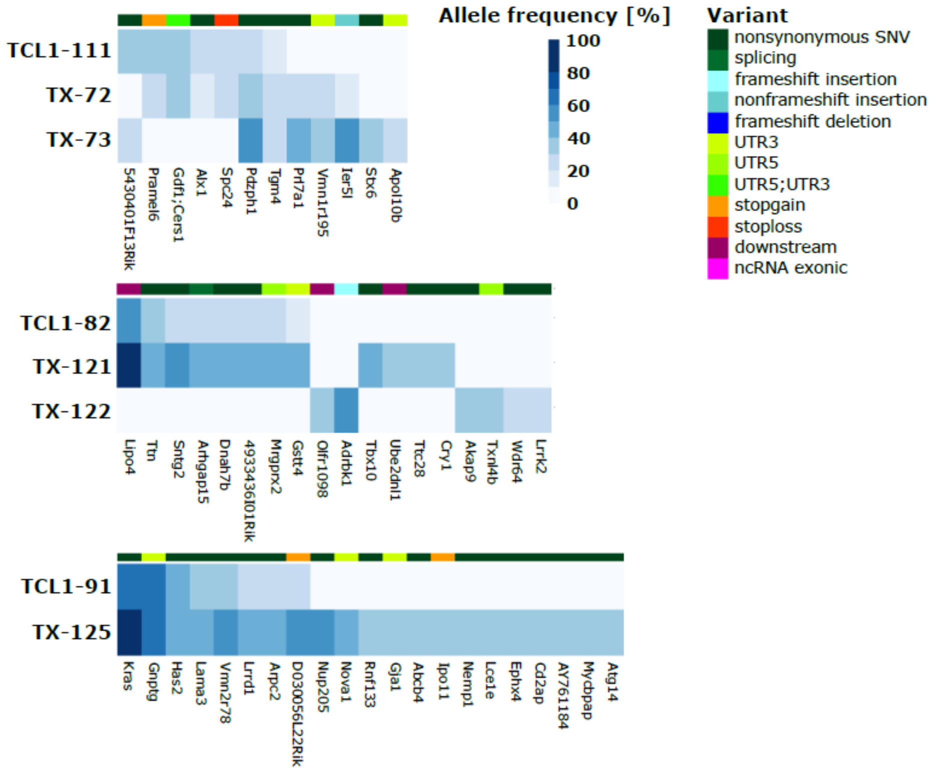

Figure S5: Somatic mutations in AID knockout TCL1 mice (TCL1-AIDKO) and congenic recipient mice (TCL1-AIDKO Tx). Heatmaps of somatic mutations found by WES of splenic CLL cells of three TCL1-AIDKO tumor lines (111, 82, 91) each transplanted into 2 congenic recipient mice (TCL1-AIDKO Tx). For line 91, only one recipient mouse developed CLL and was sequenced. Color scheme corresponds to allele frequencies of mutations. Different

classes of of somatic mutations (variant) are depicted as color scheme above the heatmap, gene names are shown below. Synonymous exonic mutations and intronic mutations are not depicted.

Supplementary figure 6

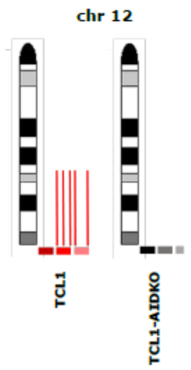

Figure S6: Chromosomal deletion on chr12 in TCL1 tumors. Length of chromosomal deletion on chr12 is shown as vertical bar present in TCL1 tumors, but absent in TCL1-AIDKO. Bar length corresponds to length of the CNV for individual mice. Horizontal bars correspond to transplantation lines (black: 82, grey: 111, light grey: 91, dark red: 347, red: D22, light red: E31) with TCL1 and Tx.

Supplementary figure 7

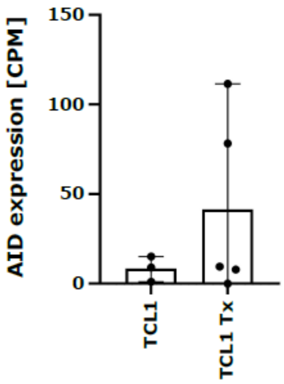

Figure S7: AID expression of sorted tumors from 3 TCL1 and 5 TCL1 Tx mice depicted as counts per million reads (CPM).

Supplementary figure 8

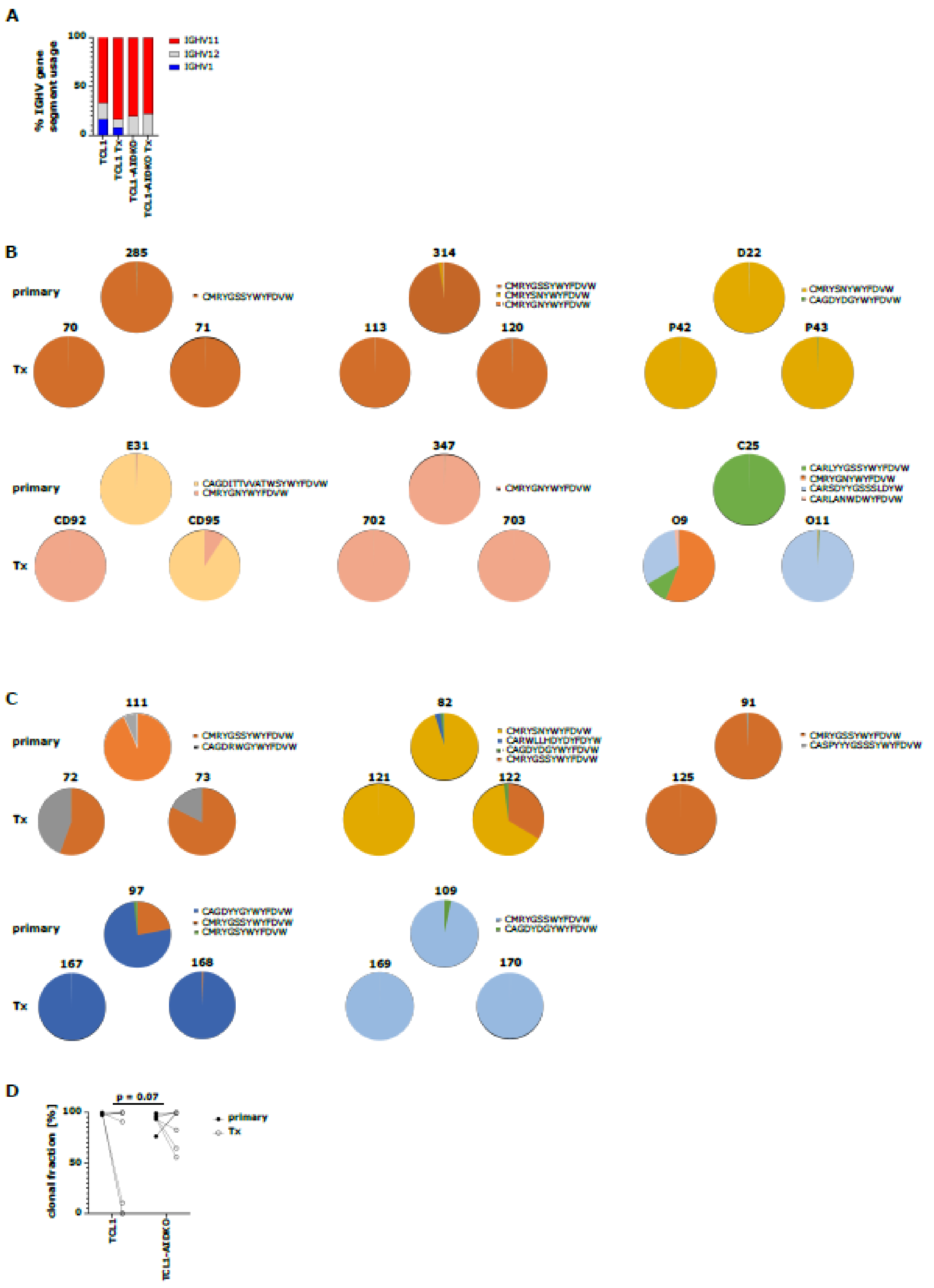

Figure S8: IGHV gene segment usage, tumor BCR clonality and clonal switch analysis. (A) Percentage of used IGHV gene segments are depicted for the major BCR clones of TCL1 (n = 6), TCL1-AIDKO (n = 5), TCL1 Tx (n = 12) and TCL1-AIDKO Tx (n = 9) mice. Pie charts depicting the frequency of specific BCR clones for primary (top) and

Tx (bottom) mice of (B) TCL1 and (C) TCL1-AIDKO tumors. The translated amino acid sequence of the CDR3 regions of IGHV is depicted in the legends. (D) Clonal switch analysis depicting the clonal fraction of the major BCR clone in the primary tumor (primary) and the same BCR clone in the transplants (Tx) for TCL1 and TCL1-AIDKO tumors. (Barnard's test: $p=0.1328$ (two-sided), $p=0.0701$ (one-sided)).
